# Horizontal Gene Transfers Link a Human MRSA Pathogen to Contagious Bovine Mastitis Bacteria

**DOI:** 10.1371/journal.pone.0003074

**Published:** 2008-08-27

**Authors:** Thomas Brody, Amarendra S. Yavatkar, Yong Lin, Jermaine Ross, Alexander Kuzin, Mukta Kundu, Yang Fann, Ward F. Odenwald

**Affiliations:** 1 The Neural Cell-Fate Determinants Section, National Institutes of Health (NIH), Bethesda, Maryland, United States of America; 2 The Division of Intramural Research Information Technology Program, National Institute of Neurological Disorders and Stroke (NINDS), National Institutes of Health (NIH), Bethesda, Maryland, United States of America; Max Planck Institute for Evolutionary Anthropology, Germany

## Abstract

**Background:**

Acquisition of virulence factors and antibiotic resistance by many clinically important bacteria can be traced to horizontal gene transfer (HGT) between related or evolutionarily distant microflora. Comparative genomic analysis has become an important tool for identifying HGT DNA in emerging pathogens. We have adapted the multi-genome alignment tool *EvoPrinter* to facilitate discovery of HGT DNA sequences within bacterial genomes and within their mobile genetic elements.

**Principal Findings:**

*EvoPrinter* analysis of 13 different *Staphylococcus aureus* genomes revealed that one of the human isolates, the hospital epidemic methicillin-resistant MRSA252 strain, uniquely shares multiple putative HGT DNA sequences with different causative agents of bovine mastitis that are not found in the other human *S. aureus* isolates. MRSA252 shares over 14 different DNA sequence blocks with the bovine mastitis ET3 *S. aureus* strain RF122, and many of the HGT DNAs encode virulence factors. *EvoPrinter* analysis of the MRSA252 chromosome also uncovered virulence-factor encoding HGT events with the genome of *Listeria monocytogenes* and a *Staphylococcus saprophyticus* associated plasmid. Both bacteria are also causal agents of contagious bovine mastitis.

**Conclusions:**

*EvoPrinter* analysis reveals that the human MRSA252 strain uniquely shares multiple DNA sequence blocks with different causative agents of bovine mastitis, suggesting that HGT events may be occurring between these pathogens. These findings have important implications with regard to animal husbandry practices that inadvertently enhance the contact of human and livestock bacterial pathogens.

## Introduction

*Staphylococcus aureus* (*S. aureus*) infections in both man and domestic livestock present growing and formidable global challenges for human and animal health concerns. Methicillin-resistant *S. aureus* (MRSA) is now the leading cause of hospital- and community-acquired *S. aureus* infections [1], [2]. Likewise, among dairy herds *S. aureus* is one of the major causal agents of contagious bovine mastitis [3], [4]. A recent survey of mastitis outbreaks in Canadian dairy cows reported a total of 3,149 *S. aureus* infections in 106 farms over the course of a single year [5].

Comparative analysis of different *S. aureus* genomes has revealed that many strains have independently acquired genes from members of their surrounding microflora that confer antibiotic resistance and/or encode virulence factors [6], [7], [8]. Horizontal gene transfer (HGT) among bacteria and their mobile genetic elements (MGEs) is the primary mode for the spread of antibiotic resistance and virulence factors in clinically important pathogens [6], [9], [10]. Bacteriophage, plasmids, transposons and uptake of naked DNA have been shown to be involved in the movement of DNA between different bacteria [7], [11], [12], [13], [14].

HGT events have played a prominent role in the rapid acquisition of antibiotic resistance in *S. aureus*. Before the emergence of methicillin resistance in the early 1960s, a steady increase in penicillin resistance, mediated by plasmid transfer, was detected in hospital infections of *S. aureus* in both the UK and the USA [8], [15]. Soon after methicillin was used in 1960, methicillin-resistant isolates were reported, and by 1967, multidrug-resistant MRSA was reported in numerous countries. Although the origins of the *mecA* gene (the principle component of methicillin resistance in *staphylococci*) are unknown, a *mecA* homologue (88% similarity) is ubiquitous in the antibiotic-susceptible *S. sciuri*, and may be a possible evolutionary precursor of the *mecA* gene of the MRSA strains. Transfer of *mecA* is mediated by the Staphylococcal Cassette Chromosome *mec* (SCC*mec*) [16].

Bacterial co-infections are likely to be a major point of origin for HGT events. For example, the gene encoding the biofilm-associated protein (Bap), a surface protein implicated in formation of aggregates of microorganisms, is present in *S. aureus* that have been isolated from chronic bovine mastitis infections, and is thought to be acquired by HGT, since it is shared by other causative agents of mastitis [17]. HGT has also been documented between different bacterial genera; transfer of vancomycin resistance has been shown to occur between *Enterococcus faecalis* and *S. aureus* [18]. Other examples of transfer under conditions of co-infection include the transfer of antibiotic resistance from *E. coli* to the plague bacillis, *Yersinia pestis* [19], and transfer of vancomycin resistance from porcine to human *Enterococcus faecium* [20].

Multi-genome alignments play an important role in the identification of unique or uniquely shared HGT DNA sequences among related or evolutionary distant bacteria, and hence can be used to detect more recently acquired virulence factor genes in emerging pathogens [10]. To expedite the search for HGT sequences, we have adapted the web-accessed comparative genomics tool *EvoPrinter* for the rapid screening of chromosomal and MGE DNA [21], [22]. *Evoprinter* works rapidly: the alignment time for a 40 kb sequence to 17 staphylococcal genomes is accomplished in less than 20 sec. *EvoPrinter* algorithms serve as an initial search tool to help identify DNA sequences that are not uniformly shared by other related bacteria. Whereas BLAST sequence homology searches highlight sequence similarities, bacterial *EvoPrinter* algorithms highlight sequence differences between DNAs that may otherwise go unnoticed in a BLAST search, especially when large sequence files are searched. The uninterrupted *EvoPrinter* readouts allow for rapid visual screening of up to 40 kb of DNA, without having to sort through the multiple pairwise BLAST alignments that include both orthologs and less related sequence comparisons. *EvoPrinter* is currently formatted for the automated comparative analysis of 17 staphylococcal, 20 streptococcal and 22 enteric bacterial genomes and can be used to detect HGT sequences among bacterial chromosomes and their MGEs. Staphylococcal genomes included in the *EvoPrinter* automated analysis currently include 13 *S. aureus*, two *S. epidermidis*, one *S. haemolyticus* and one *S. saprophyticus*.

Our search for HGT DNA sequences within the genomes of different human MRSA isolates has led to the discovery that one of the human strains, the hospital-acquired epidemic MRSA252 [23], uniquely shares multiple DNA sequence blocks with three different causative agents of contagious bovine mastitis but not with other human isolates. These putative HGT sequences encode virulence factors that are also incorporated into the chromosomes or plasmids of the bovine *S. aureus* ET3 strain RF122, *S. saprophyticus* and *Listeria monocytogenes*. Analysis of the MRSA252 complete genome *EvoUnique* profile (compared to 12 other *S. aureus* isolates), identified over 20 different regions that were either unique to the MRSA252 genome or uniquely shared with just one other *S. aureus* isolate included in the analysis, the bovine RF122. Taken together, the multiple uniquely shared DNAs indicate that the human MRSA252 or another related epidemic MRSA strain may have undergone repeated HGT events with different bovine pathogens.

## Results and Discussion

The comparative analysis of the bacterial chromosomes and their MGEs described in this study was performed using the *EvoPrinterHD* alignment algorithms. *Evoprinter* functions as a tool for the rapid discovery of unique DNA sequence differences among multiple bacterial genomes and their MGEs. Specifically, an *EvoDifference* profile identifies sequences present in a group of related genomes that are lacking in a single genome, and an *EvoUnique* profile highlights sequences that are either unique or uniquely shared by a subset of bacteria included in the analysis.

**Figure 1:**
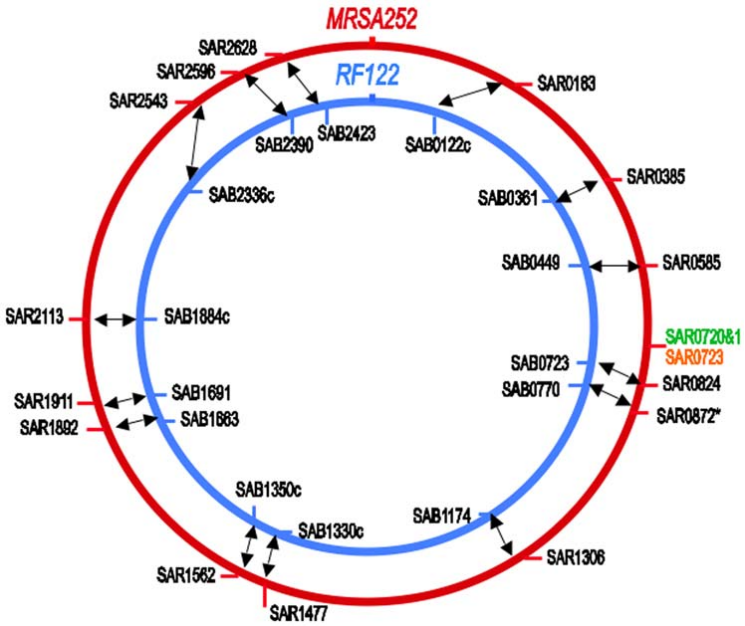
Circular displays of the *S. aureus* human MRSA252 and bovine RF122 chromosomes showing the relative genomic positions of the uniquely shared DNAs. Shown are the relative positions of the MRSA252 (SAR) and RF122 (SAB) shared genes. The chromosomal position of the MRSA252 - *Listeria monocytogenes* shared genes copper ATPase and copper oxidase is indicated in green. The chromosome position of the cadmium-transporting ATPase *cadA* gene shared with the *S. saprophyticus* pSSP2 plasmid is indicated in orange. Arrows indicate orthologous DNAs.

To identify uniquely shared DNA sequence blocks and unique single nucleotide polymorphisms (SNPs) among subsets of 13 different *S. aureus* genomes, we generated *EvoUnique* profiles of their chromosomes. Our initial MRSA252 *EvoUnique* profile alerted us to multiple instances of exclusive sequence sharing with other *S. aureus* chromosomes, indicated by green uppercase letters (Figure 1). To our surprise, the MRSA252 *EvoUnique* profile revealed that the highlighted DNAs were almost exclusively shared with a contagious bovine mastitis strain known as RF122. Notably, MRSA252 and RF122 chromosomes share 14 different unique DNA sequence blocks that are not present in any of the other *S. aureus* or in any of the other *Staphylococci* included in the *EvoPrinter* database (Figure 1, Table 1 and Table S1; see materials and methods for complete list of genomes). The different sequence blocks, which are from 144 to 4,950 bp in length, exhibit between 93% and 99% pairwise identity, with most having 97% or greater identity. Complete *EvoUnique* profiles of the MRSA252 and RF122 chromosomes are available at the *EvoPrinter* website.

**Table 1:** MRSA252 uniquely shares multiple DNA sequences with other causative agents of bovine mastitis.

Uniquely shared sequences		Sequence description	Flanking MGE or phage DNA
**MRSA252**	**RF122**		
SAR0183	SAB0122c	Partial sequence of Acetylglutamate Kinase.	None detected
SAR0385	SAB0361	Similar to bovine pathogenicity island protein ORF3	Phage
SAR0585	SAB0449	Hypothetical Phosphomethylpyrimidine kinase	None detected
SAR0824	SAB0723	Malolactic enzyme ORF plus flanking sequences.	None detected
SAR0872*	SAB0770*	Intragenic	None detected
SAR1306	SAB1174 & SAB1175c	Hypothetical novel protein.	Transposon
SAR1477 & SAR1478	SAB1330c & SAB1331c	Chorismate Synthase gene & Nucleoside Diphosphate Kinase	None detected
SAR1562*	SAB1350c & SAB1349c	Hypothetical novel proteins	Phage
SAR1892 & SAR1889	SAB1663 & others	Hyaluronate lyase precursor 1 & other hypothetical proteins	None detected
SAR1911	SAB1691	Hypothetical novel protein	None detected
SAR2113	SAB1884c	Hypothetical novel protein	Phage
SAR2543	SAB2336c	ATP binding ABC transporter	None detected
SAR2596	SAB2390	Fructose 1,6 bisphosphatase	None detected
SAR2628	SAB2423	Putative ATP-dependent protease ATP-binding subunit ClpL	None detected
**MRSA252**	***Listeria monocytogenes***		
SAR0720	FSL R2-503	*CopB* (Copper cation ATPase transporter)	Transposon
SAR0721	FSL R2-503	Copper oxidase	Transposon
**MRSA252**	***S. saprophyticus*** ** PSSP2 plasmid**		
SAR0723	SSPP217	Cadmium-transporting ATPase	Transposon

* Sequence flanks the gene.

Considerable information has been acquired about the biology of these two pathogens [23], [24], [25], [27], [28]. Comparative analysis of the human and bovine *S. aureus* strains has revealed that they are phylogenetically distinct from one another and from other *S. aureus* strains [23], [25] and that their pathogenicity-associated genes have undergone significant genetic divergence [23], [24], [25]. MRSA252 is a sub-clone of the hospital epidemic EMRSA-16 clone [23]. The EMRSA-16 clone and its representative members are responsible for half of all MRSA infections in the U.K., and it is now considered one of the most clinically important global lineages within the U.S. [23]. The MRSA252 chromosome contains a 58.8 kb SCCmec element that carries multiple antibiotic resistance genes [23]. MRSA252 also contains the Tn552 transposon that harbors penicillin-resistance genes, which are components of the inducible *S. aureus β*-lactamase operon [26]. The RF122 isolate belongs to the ST151 sub-clone of the bovine ET3 clone [27], and members of this subclone display greater virulence than other ET3 sub-clones in a mouse model of mastitis [28]. In addition, comparative analysis of the different bovine ET3 clonal subtypes revealed that multiple episodes of HGT may have occurred within the ET3 lineage [28].

**Figure 2:**
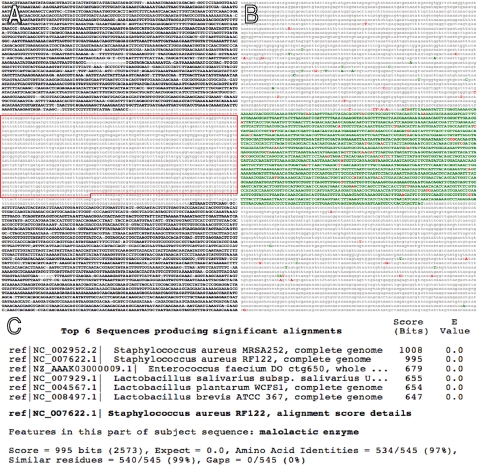
*EvoPrint* and *EvoUnique* comparative analysis of 13 different *S. aureus* isolates reveals that only the human MRSA252 and contagious bovine mastitis RF122 chromosomes contain the malolactic enzyme gene. A) An *EvoPrint* generated from pairwise alignments of MRSA252 DNA (6,497 bp) and aligning regions from 12 other *S. aureus* genomes (*S. aureus* COL; *S. aureus* MSSA476, *S. aureus* Mu50; *S. aureus* MW2; *S. aureus* N315; *S. aureus* NCTC 8325; *S. aureus* RF122; *S. aureus* USA300; *S. aureus* JH1; *S. aureus* JH9; *S. aureus* Mu3 and *S. aureus* Newman) reveals high homology between all aligning regions except for a central 1,981 bp region. Uppercase black-colored letters identify MRSA252 sequences that align with all genomes and lowercase gray-colored bases indicate nucleotides that do not align with at least one of the genomes. The boxed ORF sequence encodes the 545 amino acid malolactic enzyme. B) Generated from the same analysis, the MRSA252 *EvoUnique* print indicates that only one of the 12 genomes included in the analysis contains homologous sequences that span the MRSA252 malolactic enzyme gene locus (SAR0824). Uppercase red-colored SNPs are unique to the MRSA252 sequence; green-colored letters represent bases that align with only one of the 12 genomes (*S. aureus* RF122) and lowercase gray-colored letters represent sequences that are common to two or more alignments. (C) Protein database homology searches identify the RF122 malolactic enzyme as sharing the highest identity with the MRSA252 enzyme. Note: no homologies to other malolactic enzyme encoding genes were detected in other *S. aureus* species.

Database searches reveal that many of the MRSA252-RF122 unique sequence blocks span genes that encode virulence factors, metabolic enzymes or novel protein encoding sequences found in other bacteria (Table 1). For example, among the *Staphylococcal* genomes included in this analysis, a sequence encoding the malolactic enzyme gene (annotated respectively as SAR0824 and SAB0723 in the MRSA252 and RF122 genomes) is unique to MRSA252 and RF122 but present in many fermentation bacteria (Figure 2 and Table 1). The shared sequence, consisting of 1,981 bp, is located at synonymous genomic locations and exhibits 97.8% sequence identity (Figures 1 and 2). Malolactic enzyme is a component of the anaerobic respiration pathway and confers bacterial virulence by enabling survival in the anaerobic environment of deep tissue abscesses [29]. Given that abscess formation is an important aspect of human MRSA pathogenesis [30], the acquisition of this enzyme is most likely of clinical importance.

The *EvoUnique* profile of the malolactic enzyme region also demonstrates the utility of this approach for the detection of single nucleotide polymorphisms (SNPs). For example, the red-colored bases in Figure 2B represent SNPs that are unique in MRSA252 and not present in any of the other genomes analyzed, including RF122. The *EvoUnique* profile of the first 50 kb of the MRSA252 chromosome identifies 229 SNPs that distinguishes it from the other isolates (data not shown). The speed and base-pair resolution of this approach should prove invaluable when markers are sought to distinguish between different MRSA isolates.

All of the MRSA252-RF122 shared DNAs exhibit features of HGT events [6]; the acquired DNA is not found in other closely related strains and the sequences flanking the unique DNA share greater identity with the closely-related genomes (that lack the unique DNA) than they do with the putative donor genome. For example, pairwise alignments reveal that the highest shared identity with 5 kb of MRSA252 DNA flanking either side of the malolactic enzyme gene (Figure 2) is not with RF122 but with the human community-acquired MRSA MW2 isolate (data not shown). Of all of the genomes included in the analysis, the RF122 flanking DNA is least homologous to the MRSA252 flanks. The multiplicity and synonymous genomic locations of many of the unique DNAs indicate that multiple HGT events have occurred, and that homologous recombination most likely played a role in many of the integration events.

The bovine RF122 isolate or related clones may have undergone HGT events with other human *S. aureus* isolates. For example, DNA sequences between bases 404,577 and 409,784 of RF122 are exclusively shared with two human isolates, MSSA476 and MW2, but not with MRSA252 or the other *S. aureus* isolates examined. MSSA476 and MW2 are representative of community-acquired *S. aureus* strains; MSSA476 is a hyper-virulent community-acquired methicillin-susceptible strain isolated in the United Kingdom [23] and the MW2 strain is one of the major MRSA pathogens causing community-acquired infections in the mid-western region of the USA [31]. The shared sequences contain ORFs but do not encode proteins of identifiable function (data not shown).

**Figure 3:**
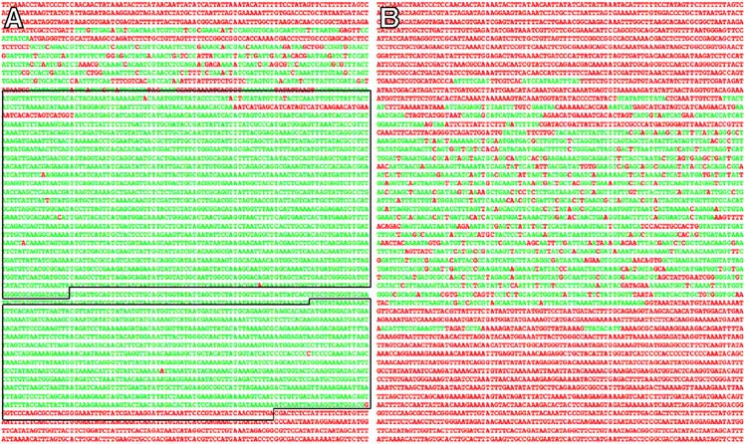
*S. aureus* MRSA252 and *Listeria monocytogenes* chromosomes share near identical copper ATPase transporter and copper oxidase genes. A) Shown is a *Listeria monocytogenes* DNA (4,680 bp) – MRSA252 pairwise enhanced BLAT (*e*BLAT) DNA alignment revealing near sequence identity that spans the ORFs for the copper ATPase transporter (SAR0720; upper box) and the copper oxidase genes (SAR0721; lower box) [23]. The *Listeria* genomic region of this *e*BLAT (GenBank accession AARR00000000), used as the reference sequence, was selected as ‘other species’ in the sequence input page of *EvoPrinter*. Use of this feature allows for the comparative analysis of any sequence with respect to the genomes in the *EvoPrinter* database. Red-colored letters indicate unique bases that not shared with MRSA252. Green-colored sequences are common to both *Listeria* and MRSA252. B) *Listeria* DNA (same sequence as in panel A) *e*BLAT alignment with the *S. aureus* USA300 genome identifies CopA (SAUSA300_0078) but with a significantly lower sequence homology when compared to the MRSA252 DNA (A).

*EvoPrinter* analysis of the MRSA252 chromosome also identified additional DNA sequence blocks that are absent from the other *S. aureus* but shared with other causative agents of bovine mastitis: *Listeria monocytogenes* and *S. saprophyticus* [32], [33]. Most notable is a region (at position 754,883) that spans the copper cation ATPase transporter *copB* (SAR0720) and copper oxidase genes (SAR0721) (Figure 1 and 3A). The MRSA252 genomic region that spans these two genes is nearly identical to DNA present in the genome of *Listeria monocytogenes*, as revealed by a BLASTn alignment (data not shown). Pairwise alignments between the *Listeria* DNA and the aligning region of the MRSA252 chromosome reveal that the two DNAs share 99.8% identity over 3,131 bp (Figure 3A). Our analysis also uncovered another human MRSA isolate, USA300, that contains a copper ATPase transporter gene (SAUSA300_0078, identified as *copA*), however, its sequence homology to the MRSA252/*Listeria* genes is significantly less and it lacks the flanking copper oxidase gene (Figure 3B). The MRSA252 *copB* and copper oxidase genes were most likely transferred via a plasmid, as the flanking DNA in MRSA252 is homologous to plasmid sequences (data not shown). The *copA* and copper oxidase genes constitute a copper resistance operon (cop) associated with resistance to metal toxicity [34]. It is surprising that *Listeria* and MRSA252 have near identical copper resistance genes. Metal ion transporters play important roles in both nutrient uptake and in secretion of toxins [35].

Another MRSA252 unique sequence block (2,152 bp; SAR0723) is also shared with the *Staphylococcus saprophyticus* plasmid pSSP2 (locus tag: SSPP217, GenBank accession NC_007352) and it encodes the cadmium-transporting ATPase CadA protein (93.5% shared identity). Heavy metal cadmium resistance has been associated with *S. aureus* plasmids [36], although the current selective pressure for cadmium resistance is unknown [37].

Our comparative analysis identified additional putative HGT DNA sequences within the MRSA252 genome that have no counterpart in other *Staphylococci* examined in this study. For example, the unique DNA sequence found at nucleotides 303,347–306,097 contains an ORF coding for a 763 amino acid protein (Figure S1), annotated as a nitric oxide reductase (SAR0261) and identified as a region of inserted DNA [23]. A BLASTp database search revealed 51% amino acid identity to nitric oxide reductase encoding genes in *Geobacillus* species and *Bacillus licheniformi* (data not shown). In other bacterial pathogens, the nitric oxide reductase enzyme is considered a virulence factor that allows survival under very low oxygen tension and/or allows the organism to take advantage of de-nitrification to cope with nitric oxide production in macrophages [38], [39].

The presence of mobile genetic element-associated genes integrated into the MRSA252 genome and the sharing of sequences between MRSA252 and RF122 prompted us to examine sequenced *S. aureus* associated MGEs for the presence of genes that might be shared by these two isolates, thus suggesting a potential mode of transfer. *EvoPrinter* analysis of the 13 different *S. aureus* isolates was performed using the 35 kb pTZ2162 plasmid as the reference input sequence. This plasmid is widely distributed among healthcare-associated MRSA strains [40]. The pTZ2162 *EvoUnique* profile identified multiple regions that are uniquely shared with different *S. aureus* genomes (data not shown). An *EvoDifference* analysis of one such region identified two flanking sub-regions, one shared with MRSA252 and the other with RF122 (Figure S2). The shared pTZ2162-MRSA252 sequence contains a partial sequence that matches the *blaZ* antibiotic resistance gene, which encodes ß-lactamase [41]. *blaZ* has been identified in a significant fraction of clinical *S. aureus* isolates [42] and in *S. aureus* isolates from persistent bovine mastitis [43]. The RF122 shared sequence contains an ORF that encodes a quinone oxidoreductase/DT diaphorase, a member of a subfamily of alcohol dehydrogenases, annotated as SAB1296c [24]. In *E. coli*, oxidoreductases have been shown to be drug resistance factors [44]. The HGT events were most likely due to plasmid insertions, as both the MRSA252 (located at 866,929) and RF122 (located at 1,418,565 bp) sequences are flanked by plasmid sequences (data not shown). The pTZ2162 plasmid also shares near sequence identity to multiple genes in *Staphylococcus haemolyticus* and *Staphylococcus epidermidis* human pathogens (data not shown).

*EvoUnique* profiles of other *S. aureus* isolates also identified putative HGT sequences that are not part of the MRSA252 or RF122 chromosomes. For example, our analysis revealed sequences within the human USA300 chromosome that are shared with *S. epidermidis*, *S. haemolyticus* and *S. saprophyticus*, but not with other *S. aureus* isolates (data not shown). Additionally, the USA300 *EvoUnique* profile revealed sequences uniquely shared with the human MW2 and Col isolates, but no sequences were found uniquely shared between USA300 and MRSA252 or RF122.

### Summary

The finding of multiple putative HGT DNAs that are shared between a human epidemic MRSA isolate and a contagious bovine mastitis *S. aureus* isolate, but that are absent from other *S. aureus*, indicates that these two phylogenetically distinct strains most likely have undergone multiple gene transfers either between themselves or with as yet unidentified additional bacteria. Clearly, comparisons between bacterial genomes establish only that they uniquely share DNA sequences, but the analysis does not establish a transfer mechanism or the identity of the exchanging partners. However, our analysis reveals multiple instances of HGT events between RF122 and MRSA252 that are adjacent to MGE and bacteriophage sequences. In most cases, the flanking vector sequences are in MRSA252 and not in RF122, suggesting that MRSA252 or one of its related clones was the recipient in the HGT exchange.

One likely scenario is that the DNA exchanges may have occurred during human and/or bovine co-infections. In light of the documented cases of direct transmission of MRSA between cows and humans [45] and the isolation in cows of a MRSA related to human epidemic strain Irish 01 [4], this possibility is highly probable. Clearly, more studies are required to determine how and where the putative HGT events took place. Nevertheless, the finding of unique virulence factor genes in a human pathogen whose potential source(s) may have originated from different causative agents of contagious bovine mastitis, including *Listeria monocytogenes* and *S. saprophyticus*, suggests that there may be a common epidemiological association between these bacteria and that co-infections are a likely point of origin for these exchanges. A similar concern regarding co-infection has been raised over the possibility of HGT of vancomycin resistance from enterococcus to *S. aureus* [46]. Taken together, these findings suggest that HGT events may be more prevalent between human and livestock bacteria than previously recognized and that animal husbandry practices that enhance contact between human and livestock pathogens should be avoided. Further work is required to elucidate the details and ramifications of these exchanges.

## Materials and Methods

### EvoPrinter analysis

*EvoPrinter* algorithms consist of a series of web-accessed tools for discovering and comparing conserved or uniquely shared sequences within orthologous DNAs [21], [22] (http://evoprinter.ninds.nih.gov/). Unlike other multi-genome comparative tools that display columns of aligning bases with gaps to optimize alignments, *EvoPrinter* displays, in a single uninterrupted view, DNA sequences that are either conserved, unique or uniquely shared, including single nucleotide polymorphisms (SNPs), as they exist in the genome or MGE of interest. Because *EvoPrinter* readouts show only the input reference DNA sequence and not the aligning regions of the multiple genomes included in the analysis, more sequence can be displayed in a single view than is possible with conventional multi-genome alignments.

The following algorithms were developed to help identify putative HGT DNA sequences: (1) an *EvoUnique* profile highlights unique or uniquely shared sequences among subsets of genomes that are otherwise absent from the other genomes included in the analysis; (2) a repeat finder detects putative MGE sequences based on the repetitive presence of their sequences within bacterial chromosomes; (3) an *EvoDifferences* profile portrays, in a single view, those sequences that are detected in all but one of the genomes included in the analysis, and (4) input reference DNA exchange allows for re-initiation of the comparative analysis using the aligning region of another genome, thus facilitating the search for unique differences among the genomes included in the analysis. *EvoPrinterHD* also includes algorithms that identify sequence rearrangements in the aligning regions of the test genomes.

For *Staphylococcus* chromosome analysis, after inserting the DNA sequence to be analyzed (the reference sequence) into the DNA sequence input window, the alignment algorithms automatically generate 9 *e*BLAT alignments for each of the 17 staphylococcal genomes and then assembles composite *e*BLAT (c*e*BLAT) alignments for the top three homology scoring regions of each genome. The alignment process takes seconds to complete and allows the user to examine the input DNA for repetitive sequences and to view the alignment results, in the form of an alignment scorecard [22]. The alignment scorecard gives information regarding the extent of homology to each of the test species for the top scoring alignment, and the second and third most significant alignments. Accessible from the alignment scorecard are the *e*BLAT alignments to the top three aligning regions for each pairwise analysis, and a c*e*BLAT that superimposes the top three alignments. From the scorecard, the user can select or deselect different alignments to be used in the *EvoPrint* analysis. The complete *EvoUnique* prints of the *S. aureus* MRSA252 and *S. aureus* RF122 genomes are also available online through links provided in the *EvoPrinterHD* bacterial genome resources section.

### Genomic DNA sequence files

The 17 *Staphylococcus* genomes were curated from databases listed below. The following *Staphylococcus* genome sequence files were curated from the BacMap database of University of Alberta (http://wishart.biology.ualberta.ca/BacMap/): *S. aureus* COL, *S. aureus* JH1, *S. aureus* JH9, *S. aureus* MRSA252, *S. aureus* MSSA476, *S. aureus* Mu3, *S. aureus* Mu50, *S. aureus* MW2, *S. aureus* N315, *S. aureus* NCTC8325, *S. aureus* Newman, *S. aureus* RF122, *S. aureus* USA300, *S. epidermidis* ATCC12228, *S. epidermidis* RP62A and *S. haemolyticus* JCSC1435. The genome sequence files for *S. aureus subsp. aureus* JH1, *S. aureus subsp. aureus* JH9, *S. aureus* Mu3, and *S. aureus subsp. aureus str.* Newman were curated from the European Bioinformatics Institute of the European Molecular Biology Laboratory (http://www.ebi.ac.uk/genomes/bacteria.html). The pTZ2162 plasmid sequence was obtained from the NCBI GenBank nucleotide sequence database.

### Genomic indexing

Each of the genomes was parsed into non-overlapping K-mers three different ways and held in memory. In addition to the original 11-mer index of BLAT [47], *EvoPrinterHD* indexes each genome into a second set of non-overlapping 11-mers, offset by four base pairs from the initial indexing, and into a third set of non-overlapping 9-mers. By performing three independent alignments using the staggered genomic indices and then superimposing the resulting alignments to show all aligning sequences, the *enhanced*-BLAT (*e*BLAT) detects as much as 75% more conserved sequences when evolutionary distant sequences are aligned [22].

### NCBI BLAST database searches

Database homology BLAST searches against 988 microbial genomes were performed using the standard tBLASTn, BLASTn and BLASTp options at the National Center for Biotechnology Information web site (http://www.ncbi.nlm.nih.gov).

## Supporting Information

Table S1 MRSA252-RF122 uniquely shared chromosomal DNA sequence blocks.
(0.06 MB DOC)

Figure S1 An EvoUnique profile identifies a putative nitric oxide reductase gene in the human S. aureus MRSA252 genome that is not present in the genomes of 16 other Staphylococci. EvoUnique analysis of the human S. aureus MRSA252 genomic DNA (bases 303,347 to 306,097) with 16 other Staphylococcus genomes (S. aureus COL; S. aureus MSSA476, S. aureus Mu50; S. aureus MW2; S. aureus N315; S. aureus NCTC 8325; S. aureus RF122; S. aureus USA300; S. aureus JH1; S. aureus JH9; S. aureus Mu3; S. aureus Newman; S. epidermidis ATCC 12228; S. epidermidis RP62; S. haemolyticus JCSC1435; and S. saprophyticus) reveals a unique 2,750 bp region that contains a 763 amino acid protein encoding ORF identified as encoding a nitric oxide reductase (SAR0261). Upper case, red-colored letters represent sequences that are unique to S. aureus MRSA252 and were not found in the other 16 Staphylococci genomes analyzed; green bases are shared with one other isolate and lowercase gray-colored bases are common to three or more of the test species aligning regions. Protein database homology searches reveal that the predicted protein shares 52% homology with the Geobacillis kaustophilus HTA426 nitric oxide reductase enzyme.
(4.89 MB TIF)

Figure S2 Identification of HGT exchanges between plasmid and bacterial genomes. EvoPrinter comparative analysis of the Staphylococcus pTZ2162 plasmid with the S. aureus COL; S. aureus MRSA252; S. aureus MSSA476, S. aureus Mu50; S. aureus MW2; S. aureus N315; S. aureus NCTC 8325; S. aureus RF122; S. aureus USA300; S. aureus JH1; S. aureus JH9; S. aureus Mu3 and S. aureus Newman genomes identifies HGT sequences that are uniquely shared with different bacterial genomes. Shown is a pTZ2162 plasmid DNA EvoDifferences profile (25,815 to 30,799 bp) that highlights two different putative HGT events. Highlighted with green-colored letters, pTZ2162 uniquely shares a 766 bp sequence with the human MRSA252 genome that includes a partial match to the blaZ gene (ORF boxed). Flanking the MRSA252 - pTZ2162 shared homology is a 2,121 bp fragment (red-colored sequences) that is uniquely shared with the bovine RF122 chromosome that contains the quinone oxidoreductase / DT diaphorase gene (ORF boxed).
(5.06 MB TIF)
