# Supplementary material for: Horizontal Gene Transfers Link a Human MRSA Pathogen to Contagious Bovine Mastitis Bacteria
Source: PLoS One. 2008 Aug 27;3(8):e3074. doi: 10.1371/journal.pone.0003074 (PMC2518619; doi:10.1371/journal.pone.0003074)
Supplement: Table S1 — MRSA252-RF122 uniquely shared chromosomal DNA sequence blocks. (0.06 MB DOC) [file pone.0003074.s001.doc]

| **Table S1. MRSA252-RF122 uniquely shared chromosomal DNA sequence blocks** | | | | | |
| --- | --- | --- | --- | --- | --- |
| **Human MRSA252 Genomic Position^*^** | **Bovine RF122 Genomic Position^*^** | **Length of Core**  **Shared DNA** | **% Identity NT/AA** | **MRSA252 & RF122 gene identity** | **Gene/Comments** |
| 207,207 - 207,339 | 150,894 - 151,026 | 133 bp | 97/98 | SAR0183**  SAB0122c | Sequence includes 3’ end of an ORF encoding a probable acetylglutamate kinase. NFS**^‡^** |
| 422,867 - 423,549 | 401,274 - 401,953 | 683 bp | 99/99 | SAR0385  SAB0361 | Sequence includes the Bovine Pathogenicity Island ORF3. MRSA252 flanking sequence has poor match to bacteriophage Orf2 |
| 634,543 - 634,876 | 594,982 - 595,315 | 334 bp | 94/99 | SAR0585  SAB0449 | Sequence includes an ORF encoding a hypothetical Phosphomethylpyrimidine Kinase (276 aa). NFS |
| 866,806 - 868,771 | 794,798 - 796,756 | 1,981 bp | 98/98 | SAR0824  SAB0723 | Sequence includes all of the Malolactic Enzyme ORF plus flanking sequences. NFS |
| 911,148 - 911,345 | 840,290 - 840,485 | 197 bp | 96/none | SAR0872**^‡‡^**  SAB0770**^‡‡^** | Intergenic sequence located 3’ to a putative Lipoprotein gene (SAR0872). NFS |
| 1,368,849 - 1,369,674 | 1,279,384 - 1,280,109 | 725 bp | 97/97 | SAR1306  SAB1174 & SAB1175c | Sequence encodes a hypothetical novel protein. MRSA252 sequence is flanked by a bacterial transposase |
| 1,572,310 - 1,572,449 | 1,460,001 - 1,460,139 | 140 bp | 94/99 | SAR1477 & SAR1478  SAB1330c &SAB1331c | Sequence includes 5' end of the Chorismate Synthase gene and 3' flank of a Nucleoside Diphosphate Kinase. NFS |
| 1,637,612 - 1,639,437 | 1,478,398 - 1,480,222 | 1,825 bp | 97/99 | SAR1562  SAB1350c & SAB1349c | Two hypothetical proteins in RF122, one listed as probable lipoprotein. MRSA252 sequence is flanked a phage integrase |
| 1,973,119 - 1,977,526 | 1,801,131 - 1,805,984 | 4408 bp | 93/90 | SAR1892 & SAR1889  SAB1663 & others | Hyaluronate lyase precursor 1 and a hypothetical genes. and other hypothetical genes NFS |
| 1,990,186 - 1,993,685 | 1,833,367 - 1,836,866 | 3,500 bp | 97/97 | SAR1911  SAB1691 | Hypothetical protein (448 aa) and other hypothetical protein. NFS |
| 2,175,265 - 2,176,819 | 2,020,265 - 2,021,820 | 1,556 bp | 98/97 | SAR2113  SAB1884c | RF122 sequence is a hypothetical phage-related protein. Flanking sequence gives poor match to transposase |
| 2,625,057 - 2,626,309 | 2,472,431 - 2,473,691 | 1,261 bp | 97/99 | SAR2543  SAB2336c | ATP-binding ABC transporter NFS |
| 2,680,822 - 2,683,412 | 2,525,624 - 2,528,115 | 2,642 bp | 98/99 | SAR2596  SAB2390 | Fructose 1,6-bisphosphatase NFS |
| 2,712,518 - 2,714,978 | 2,558,277 - 2,560,747 | 2,475 bp | 97/98 | SAR2628  SAB2423 | Putative ATP-dependent protease ATP-binding subunit ClpL (702 aa). NFS |
| ***Denotes base-pair positions within the complete genome**  **** SAR and SAB numbers denote defined MRSA252 and RF122 genes respectively**  **‡ NFS – Flanking sequence does not contain evidence of phage or plasmid transfer.**  **‡‡ Sequence flanks gene** | | | | | |
