# Supplementary figures and images for: Horizontal Gene Transfers Link a Human MRSA Pathogen to Contagious Bovine Mastitis Bacteria
Source: PLoS One. 2008 Aug 27;3(8):e3074. doi: 10.1371/journal.pone.0003074 (PMC2518619; doi:10.1371/journal.pone.0003074)

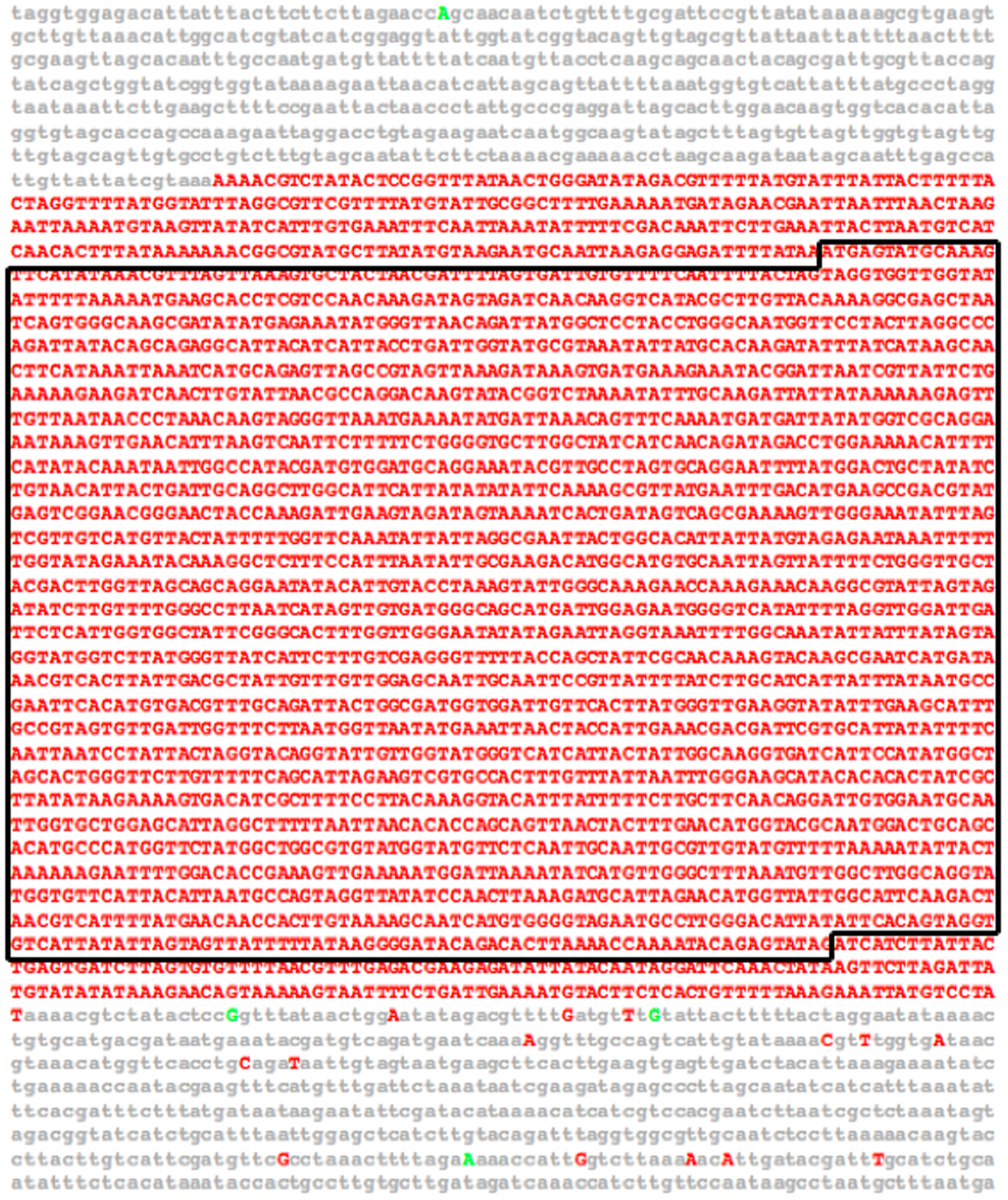

Supplement: Figure S1 — An EvoUnique profile identifies a putative nitric oxide reductase gene in the human S. aureus MRSA252 genome that is not present in the genomes of 16 other Staphylococci. EvoUnique analysis of the human S. aureus MRSA252 genomic DNA (bases 303,347 to 306,097) with 16 other Staphylococcus genomes (S. aureus COL; S. aureus MSSA476, S. aureus Mu50; S. aureus MW2; S. aureus N315; S. aureus NCTC 8325; S. aureus RF122; S. aureus USA300; S. aureus JH1; S. aureus JH9; S. aureus Mu3; S. aureus Newman; S. epidermidis ATCC 12228; S. epidermidis RP62; S. haemolyticus JCSC1435; and S. saprophyticus) reveals a unique 2,750 bp region that contains a 763 amino acid protein encoding ORF identified as encoding a nitric oxide reductase (SAR0261). Upper case, red-colored letters represent sequences that are unique to S. aureus MRSA252 and were not found in the other 16 Staphylococci genomes analyzed; green bases are shared with one other isolate and lowercase gray-colored bases are common to three or more of the test species aligning regions. Protein database homology searches reveal that the predicted protein shares 52% homology with the Geobacillis kaustophilus HTA426 nitric oxide reductase enzyme. (4.89 MB TIF) [file pone.0003074.s002.tif]

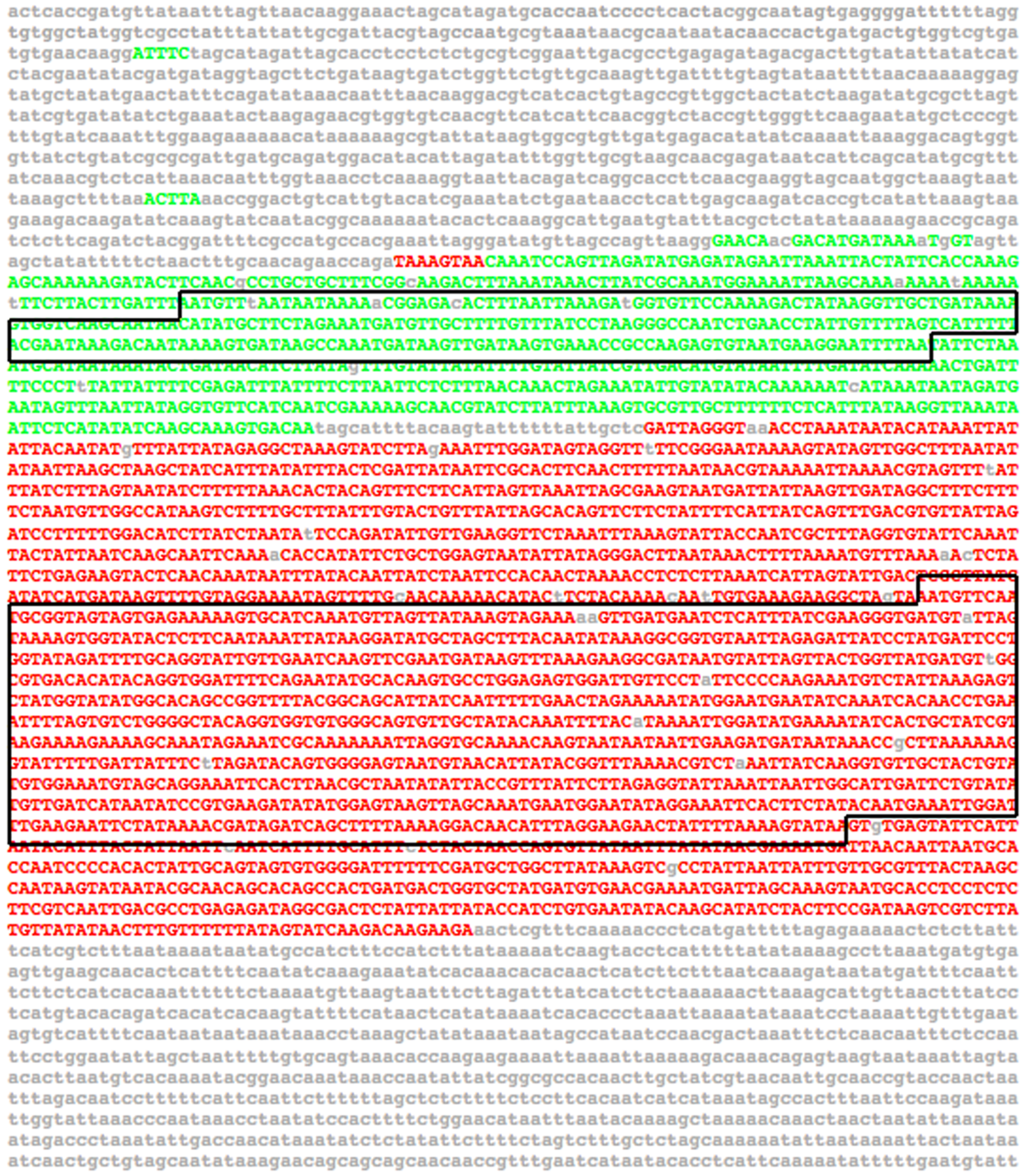

Supplement: Figure S2 — Identification of HGT exchanges between plasmid and bacterial genomes. EvoPrinter comparative analysis of the Staphylococcus pTZ2162 plasmid with the S. aureus COL; S. aureus MRSA252; S. aureus MSSA476, S. aureus Mu50; S. aureus MW2; S. aureus N315; S. aureus NCTC 8325; S. aureus RF122; S. aureus USA300; S. aureus JH1; S. aureus JH9; S. aureus Mu3 and S. aureus Newman genomes identifies HGT sequences that are uniquely shared with different bacterial genomes. Shown is a pTZ2162 plasmid DNA EvoDifferences profile (25,815 to 30,799 bp) that highlights two different putative HGT events. Highlighted with green-colored letters, pTZ2162 uniquely shares a 766 bp sequence with the human MRSA252 genome that includes a partial match to the blaZ gene (ORF boxed). Flanking the MRSA252 - pTZ2162 shared homology is a 2,121 bp fragment (red-colored sequences) that is uniquely shared with the bovine RF122 chromosome that contains the quinone oxidoreductase / DT diaphorase gene (ORF boxed). (5.06 MB TIF) [file pone.0003074.s003.tif]
